# COVID-19 cumulative incidence, asymptomatic infections, and fatality in Long Island, NY, January–August 2020: A cohort of World Trade Center responders

**DOI:** 10.1371/journal.pone.0254713

**Published:** 2021-07-20

**Authors:** Olga Morozova, Sean A. P. Clouston, Jennifer Valentine, Alexander Newman, Melissa Carr, Benjamin J. Luft

**Affiliations:** 1 Department of Family, Population and Preventive Medicine, Renaissance School of Medicine, Stony Brook University (SUNY), Stony Brook, New York, United States of America; 2 Program in Public Health, Stony Brook University (SUNY), Stony Brook, New York, United States of America; 3 World Trade Center Health and Wellness Program, Stony Brook University (SUNY), Stony Brook, New York, United States of America; 4 Department of Medicine, Renaissance School of Medicine, Stony Brook University (SUNY), Stony Brook, New York, United States of America; Seoul National University College of Medicine, REPUBLIC OF KOREA

## Abstract

**Background:**

New York City and Long Island, NY were early foci of the COVID-19 epidemic in the US. The effects of COVID-19 on different sub-populations, and its key epidemiologic parameters remain unknown or highly uncertain. We investigated the epidemiology of COVID-19 from January to August of 2020 in an established academic monitoring cohort of N = 9,697 middle-aged World Trade Center responders residing in Long Island, NY.

**Methods:**

A seroprevalence survey and a series of cross-sectional surveys were nested in a prospective cohort study. Measures included IgG antibody testing, SARS-CoV-2 polymerase chain reaction (PCR) testing, review of electronic medical records, and surveys of symptoms. Correlates of infection were analyzed with multivariable logistic regression.

**Results:**

The cohort was predominantly men in their mid-fifties; 6,597 cohort members were successfully contacted (68%); 1,042 (11%) individuals participated in the seroprevalence survey; and 369 individuals (5.6% of 6,597 study participants) underwent PCR testing. The estimated standardized cumulative incidence was 21.9% (95%CI: 20.1–23.9%), the asymptomatic proportion was 16.4% (36/219; 95%CI: 11.8–22.0%), the case hospitalization ratio was 9.4% (36/385; 95%CI: 6.6–12.7%), the case fatality ratio was 1.8% (7/385; 95%CI: 0.7–3.7%), and the hospitalization fatality ratio was 8.3% (3/36; 95%CI: 1.8–22.5%). Confirmed SARS-CoV-2 infection was associated with younger age, race/ethnicity, and being currently employed.

**Conclusions:**

The results of the present study suggest a high cumulative incidence of SARS-CoV-2 among WTC responders in the spring and summer of 2020 and contribute to narrowing the plausible range of the proportion of infections that exhibit no symptoms. An increased risk of infection among younger employed individuals is likely to reflect a higher probability of exposure to the virus, and the racial disparities in the infection risk warrant further investigation.

## Introduction

Since late 2019, SARS-CoV-2, the causative agent of the respiratory disease called COVID-19, has caused a global pandemic, with New York City (NYC) being an early focus of the outbreak in the US [1]. Long Island, NY, where essential workers for NYC often reside, was also affected early in the epidemic, with large numbers of cases and deaths attributed to COVID-19 [2, 3]. The first confirmed cases of COVID-19 were detected on March 1^st^, 2020 in NYC and on March 5^th^, 2020 in Long Island. In response to an outbreak, starting on March 12^th^, the State of New York began mandatory work-from-home and social distancing orders to combat the epidemic. Several studies have estimated the seroprevalence in NYC or an average for New York State [3–6]. To our knowledge, only one study, through convenience sampling, has provided regional estimates that included Long Island [3].

A rapidly growing body of research is investigating COVID-19 symptoms, clinical features, and infectivity [7–13]. Current epidemiologic work examining and modeling infection dynamics in the US population [14] relies on estimates of basic epidemiologic parameters largely derived from reported cases (e.g. [15, 16]). Population-level seroprevalence studies conducted early in the epidemic [4, 17–20] have suggested low infection ascertainment rates, although some of these studies might have had selection bias. Several key epidemiologic parameters—particularly the proportions of cases that are asymptomatic and cases that were severe and required hospitalization—remain highly uncertain. A recent review has found estimates of the asymptomatic fraction ranging between 6% and 96% [21]. Given the high variability in the available estimates, concerns have been raised regarding the methods in many of these studies [22]. How the asymptomatic proportion varies depending on demographic characteristics and comorbid conditions remains unclear. Several studies have estimated the proportion of cases categorized as severe [15, 23], mostly among confirmed cases in China, and have provided results that may not be generalizable to other countries [24, 25]. Although studies have been conducted among residents of congregate settings [26–28], healthcare workers [29], and patients with specific conditions [30, 31], the effects of COVID-19 on non-institutionalized and non-hospitalized populations remain largely unknown.

To fill these knowledge gaps, we used a unique opportunity to investigate COVID-19 epidemiology in an established academic monitoring cohort of World Trade Center (WTC) responders residing in Long Island, NY [32]. The primary goals of the present study were 1) to estimate the cumulative incidence of SARS-CoV-2 between the epidemic onset (mid-February) and August 2020, and 2) to estimate the asymptomatic proportion, case hospitalization ratio (CHR), case fatality ratio (CFR), and hospitalization fatality ratio (HFR) among the cohort of WTC responders. Our secondary goals included: 1) describing COVID-19 antibody response in a nested seroprevalence survey; 2) identifying correlates of infection, asymptomatic infection, hospitalization, and death in this cohort; and 3) describing the epidemic curve among symptomatic patients.

## Materials and methods

The World Trade Center Health and Wellness Program (WTC-HWP) has been described previously [33]. In brief, the WTC-HWP monitors men and women who worked or volunteered at the WTC sites during and after the tragic events of 9/11/2001. Since July 2002, WTC responders have been eligible for prospective monitoring for WTC-related conditions. Soon after the state-wide lockdown was implemented on March 12^th^, 2020, all in-person visits were cancelled, and the WTC-HWP clinic began conducting telehealth visits. With this directive, research staff made efforts to shift research activities online, thus providing an opportunity to survey cohort members regarding their COVID-19 symptoms, testing, and status.

### Sample: Seroprevalence survey

Living WTC responders participating in an ongoing monitoring program (N = 9,697) were eligible for this study. To determine cumulative incidence and the fraction of infections that did not exhibit symptoms, we conducted a seroprevalence survey by testing study participants for antibodies to SARS-CoV-2 by using the Abbott Architect IgG antibody assay (sensitivity = 93.4% 21 days after symptom onset and specificity = 100%) [34]. Testing was conducted among a consecutive sample of WTC-HWP cohort members who presented for routine annual monitoring visits and agreed to be tested. The testing dates ranged between March 25^th^ and September 18^th^, 2020, and most tests were performed in June and July. The target sample size was N = 1,000; however, some patients received antibody testing at partner laboratories, thus resulting in a final sample size of N = 1,042 patients with available antibody testing results. We provide a comparison of demographic characteristics among the seroprevalence survey sample and the remaining N = 8,655 WTC-HWP cohort members.

### Sample: Outreach surveys

To identify WTC-HWP cohort members affected by COVID-19, the entire cohort was asked, through multiple modalities including telehealth visits, in-person visits, via email and text messaging, and through direct calls, to provide information on their COVID-19 status at multiple time points between March 15^th^ and August 31^st^, 2020. First, between March 17^th^ and May 28^th^, a sample of WTC responders was seen during a comprehensive telehealth visit, at which time they were able to discuss COVID-19 symptoms (N = 1,053). Second, WTC responders were asked to respond to each of six waves of a brief needs assessment survey (N = 4,665). The survey was administered via e-mail and text messaging between March 16^th^ and June 2^nd^. Third, a brief text-based survey was sent on July 9^th^ to all WTC responders, who were asked whether they had been infected and diagnosed with COVID-19 (N = 3,417). Fourth, information was available for an additional 53 patients who neither responded to the outreach efforts listed above nor participated in the seroprevalence survey. Information about these patients’ COVID-19 history was primarily obtained during monitoring visits, during the calls made to schedule monitoring visits, and via the electronic medical records (EMR) notification system. Some patients responded multiple times to different outreach efforts, thus providing a final outreach analytic sample of N = 6,093 WTC responders (63% of eligible cohort members, hereafter the “*main outreach sample*”), of whom N = 770 cohort members also participated in the seroprevalence survey. The S1 Appendix provides details regarding the sample composition and overlap among the outreach modalities. To determine when symptoms began and whether individuals had been diagnosed or tested and when, we scheduled a standard follow-up call for all individuals who reported any symptoms [35] or diagnoses, or tested positive for antibodies. The follow-up calls were performed by trained research staff, certified medical assistants, trained case managers, or healthcare providers.

Noting that prior efforts had missed responders who ignored passive outreach efforts, potentially because they lacked symptoms, we conducted a brief telephone-based assessment in a sample of a sub-cohort of people who had not completed any outreach survey form. Among the 3,604 cohort members who had not responded to the main outreach surveys (“*non-participants*”), we selected a random sample of size N = 320, and between July 16^th^ and September 18^th^, 2020, we successfully contacted N = 255 of them, thus resulting in an 80% response rate (hereafter the “*confirmatory outreach sample*”). No statistically significant differences were observed between these responders and the remaining N = 65 individuals in terms of age, sex, race, and employment status. Among the 255 patients in the confirmatory outreach sample, 23 participated in the seroprevalence survey.

### Measures

The results of the seroprevalence survey were stratified by demographic characteristics, the history of symptoms, and the history and results of polymerase chain reaction (PCR) testing. In addition, we examined the time between the antibody testing and the onset of symptoms or PCR diagnosis, and analyzed its relationship to the antibody testing results and titers among those who tested positive. Antibody titers were available for a subset of patients who tested positive. We compared these results among patients with and without history of symptoms.

On the basis of multiple data sources, we classified all individuals as one of the following: confirmed COVID-19 positive, confirmed COVID-19 negative, symptomatic / PCR-negative / not tested for antibodies, symptomatic untested, and without known history of symptoms or testing. A case was defined as confirmed COVID-19 positive if a positive IgG antibody test or an EMR- or self-reported positive PCR test was present. A case was defined as confirmed COVID-19 negative if it was antibody-negative and was not classified as confirmed positive. Symptomatic patients with negative PCR test results and no antibody testing were classified as a separate category, because the PCR tests might have been false negative or performed outside the detection window. We show the epidemiologic curve of symptom onset dates stratified by confirmed or unconfirmed status and excluding symptomatic cases confirmed to be negative.

The COVID-19 positive cases were classified as asymptomatic, mildly symptomatic (with recovery without hospitalization), or severe (with hospitalization and/or death), on the basis of self-reported symptomatology and the EMR. Disease progression characteristics included CHR, CFR, and HFR.

### Estimation of epidemiologic parameters

We provide estimates of the following epidemiologic parameters of interest: cumulative incidence of SARS-CoV-2 through August 31, 2020, asymptomatic proportion, CHR, CFR, and HFR.

Cumulative incidence was estimated as the proportion of seroprevalence survey participants who tested positive for IgG antibodies. Given the differences in some demographic characteristics between the seroprevalence survey sample and the remaining cohort members, we used logistic regression to perform standardization of cumulative incidence according to the demographic characteristics that significantly differed between groups [36]. In addition, we computed the cumulative incidence by including survey participants who tested negative for IgG antibodies, but who had a record of a positive PCR test.

The asymptomatic proportion was estimated among the seroprevalence survey participants who tested positive for IgG antibodies. To corroborate this result, we additionally estimated the asymptomatic proportion among the confirmed positive COVID-19 cases identified via the outreach survey, adjusting for potential self-selection bias based on the stratified results estimated in the main and the confirmatory outreach samples (estimation approach details are provided in the S1 Appendix).

Beyond the COVID-19 cases identified via the seroprevalence survey, the outreach survey efforts identified another 166 confirmed positive COVID-19 cases, thus increasing the sample size used to estimate CHR, CFR, and HFR, as well as to identify correlates of confirmed infection. CHR was estimated as a proportion of all confirmed positive COVID-19 cases aggregated from seroprevalence and outreach surveys with hospitalization for COVID-19 for at least 1 day. CFR was estimated as a proportion of all confirmed positive COVID-19 cases resulting in death, and with COVID-19 listed as either a primary or a contributing cause of death. HFR was estimated in a similar manner among patients hospitalized for COVID-19.

### Correlates of infection

We examined the following characteristics as potential correlates of confirmed infection: *Demographics* including age in years, sex, race/ethnicity, and current employment status; *medical factors* including history of hypertension, diabetes, all-cause cancer, obstructive airway disease, upper respiratory disease, morbid obesity, depression, post-traumatic stress disorder (PTSD), and anxiety; and *pharmaceutical factors* including prescriptions of ibuprofen, angiotensin-converting enzyme (ACE) inhibitors, statins, and steroids. Correlates of confirmed infection were examined in the subsample of confirmed positive and confirmed negative individuals pooled from both seroprevalence and outreach surveys. Low counts of asymptomatic infections, hospitalizations, and deaths among the confirmed cases prohibited exploration of correlates of these outcomes.

### Statistical analyses

Descriptive analyses used percentages and means alongside standard deviations. Sample means were compared with independent sample t-test, and proportions were compared with Pearson’s chi-squared test (p-values calculated using bootstrap with 1,000,000 iterations [37]). The variance and 95% confidence intervals (95% CI) for proportions were calculated with finite population correction if a given sample exceeded 5% of the target population [38]. The variance for the estimates of selection bias-corrected proportions was estimated conservatively by assuming independence between the main and confirmatory outreach samples. Correlates of confirmed infection were analyzed with multivariable logistic regression. Analysis was performed in the R statistical computing environment [39].

### Ethics

The Stony Brook University Ethics Review Board approved this study (#604113). The participants provided signed written informed consent.

## Results

### Demographics

The WTC-HWP cohort (N = 9,697) comprised mostly men in their mid-fifties. Table 1 shows the demographic characteristics of the entire cohort, as well as a comparison between the seroprevalence survey sample (N = 1,042) and the remaining cohort (N = 8,655). WTC responders in the seroprevalence survey sample were slightly older and more likely to be retired than the rest of the cohort.

**Table 1:** Demographic characteristics of the World Trade Center responder cohort (N = 9,697) disaggregated by participation in the seroprevalence survey (N = 1,042 participants).

Characteristic	WTC responder cohort (N = 9,697 ^a^)		Seroprevalence survey sample (N = 1,042 ^a^)		Remainder of the cohort (N = 8,655 ^a^)		
	N	%	N	%	N	%	p-value ^b^
**Age, years**: mean (SD)	55.4 (8.5)		56.6 (8.0)		55.3 (8.6)		< 0.0001
**Age, categories, years**							
30–39	143	1.5%	4	0.4%	139	1.6%	< 0.0001
40–49	2247	23.3%	190	18.2%	2057	23.9%	
50–59	4467	46.3%	516	49.5%	3951	45.9%	
60–69	2137	22.2%	257	24.7%	1880	21.9%	
70–79	589	6.1%	72	6.9%	517	6.0%	
80+	58	0.6%	3	0.3%	55	0.6%	
**Sex**							
Male	8758	90.3%	944	90.6%	7814	90.3%	0.7812
Female	939	9.7%	98	9.4%	841	9.7%	
**Race / ethnicity**							
Non-Hispanic white	7483	77.2%	798	76.6%	6685	77.2%	< 0.0001
Black	490	5.1%	50	4.8%	440	5.1%	
Hispanic	646	6.7%	42	4.0%	604	7.0%	
Other / multiracial / unknown	1078	11.1%	152	14.6%	926	10.7%	
**Employment**							
Employed	5248	54.1%	516	49.5%	4732	54.7%	< 0.0001
Retired	3667	37.8%	477	45.8%	3190	36.9%	
Unemployed / laid off	128	1.3%	9	0.9%	119	1.4%	
On extended leave or disability	490	5.1%	28	2.7%	462	5.3%	
Other / unknown	164	1.7%	12	1.2%	152	1.8%	

^a^ Numbers may not sum to the totals because of missing values, and percentages may not sum to 100 because of rounding.

^b^ p-values for independent sample t-test or Pearson’s chi-squared test, simulated using bootstrap with 1,000,000 iterations.

S1 Table in the S1 Appendix shows the demographic characteristics of the main and confirmatory outreach samples. In brief, the main outreach sample was slightly older and more likely to be retired or unemployed.

### Seroprevalence survey results

Table 2 shows the results of the seroprevalence survey, stratified by demographic characteristics, history of COVID-19 symptoms, and PCR testing. Overall, 21% (219/1042; 95%CI: 18.6–23.6%) of the seroprevalence survey participants tested positive for IgG antibodies. The proportion testing positive was significantly higher among younger working individuals and among black and Hispanic WTC responders than non-Hispanic white responders. Among the 760 survey participants who did not have COVID-19 symptoms, 4.7% (36/760; 95%CI: 3.3–6.5%) had a positive antibody test. A total of 166 patients, 163 of whom were symptomatic, received both PCR and antibody tests, and most results were concordant. Six of 130 (4.6%) participants who had a positive PCR test had a negative antibody test, in agreement with the antibody test sensitivity (the time between the PCR and antibody tests varied between 23 and 81 days). All of them were symptomatic and recovered; one was hospitalized. Fourteen of 36 (38.9%) patients who tested negative with PCR had a positive antibody test, all of whom were mildly symptomatic. Three asymptomatic cases had concordant positive PCR and antibody testing results.

**Table 2:** Proportion of the seroprevalence survey participants (N = 1,042) testing positive for IgG antibodies to SARS-CoV-2.

Characteristic	% positive (event N / total N)	95% CI for the proportion	p-value ^a^
**All survey participants**	21.0% (219 / 1042)	18.6–23.6%	
**Age, categories, years old**			
30–49	32.0% (62 / 194)	25.5–39.0%	<0.0001
50–59	21.5% (111 / 516)	18.0–25.3%	
60–69	14.4% (37 / 257)	10.3–19.3%	
70+	12.0% (9 / 75)	5.6–21.6%	
**Sex**			
Male	20.4% (193 / 944)	17.9–23.2%	0.1918
Female	26.5% (26 / 98)	18.1–36.4%	
**Race / ethnicity**			
Non-Hispanic white	20.3% (162 / 798)	17.6–23.3%	0.0716
Black	32.0% (16 / 50)	19.5–46.7%	
Hispanic	31.0% (13 / 42)	17.6–47.1%	
Other / multiracial / unknown	18.4% (28 / 152)	12.6–25.5%	
**Employment**			
Employed	26.9% (139 / 516)	23.2–31.0%	<0.0001
Retired / unemployed / disabled	15.2% (80 / 526)	12.2–18.6%	
**Experienced COVID-19 symptoms**			
Yes	64.9% (183 / 282)	59.0–70.5%	<0.0001
No	4.7% (36 / 760)	3.3–6.5%	
**PCR testing**			
Tested positive	95.4% (124 / 130)	90.2–98.3%	<0.0001
Tested negative	38.9% (14 / 36)	23.1–56.5%	
Not tested	9.2% (81 / 876)	7.4–11.4%	

^a^ p-values for Pearson’s chi-squared test, simulated using bootstrap with 1,000,000 iterations.

Among 219 participants with a positive antibody test (IgG antibody test result > 1.0 AU/ml), titers information was available for 144 individuals: 119 symptomatic and 25 asymptomatic. The mean IgG antibody titers corresponded to 83 AU/ml (SD = 49) among symptomatic cases and 65 AU/ml (SD = 53) among asymptomatic cases (p = 0.099). In the available sample, we did not find any association between antibody positivity or titers and the time to antibody testing after symptom onset or PCR diagnosis.

### Estimates of epidemiologic parameters

#### Cumulative incidence

The proportion of seroprevalence survey participants who tested positive for IgG antibodies was 21.0% (219/1042; 95%CI: 18.6–23.6%). The cumulative incidence standardized by age (categorical variable), race, and employment status (binary variable) was similar: 21.3% (95%CI: 19.5–23.2%). As part of the outreach survey, we identified 6 seroprevalence survey participants who tested negative for IgG antibodies but had a history of a positive PCR test, in agreement with the antibody test sensitivity estimates. This adjustment increased the crude cumulative incidence to 21.6% (225/1042; 95%CI: 19.1–24.2%) and the standardized cumulative incidence to 21.9% (95%CI: 20.1–23.9%).

#### Asymptomatic proportion

The proportion of seroprevalence survey participants who did not exhibit any COVID-19 symptoms among those who tested positive for IgG antibodies was 16.4% (36/219; 95%CI: 11.8–22.0%). Among the confirmed COVID-19 cases identified among the outreach survey participants, the respective proportion adjusted for selection bias was very similar, but the confidence interval was wider because of the smaller confirmatory sample size: 16.8% (95%CI: 6.8–26.9%).

Combined seroprevalence and outreach surveys identified a total of 385 confirmed positive COVID-19 cases: 219 were identified via the seroprevalence survey, and an additional 166 cases were identified via outreach survey efforts. Because some of the outreach survey participants were also sampled in the seroprevalence study, the total number of confirmed COVID-19 cases in the outreach survey samples was 353. S2 Table in the S1 Appendix shows SARS-CoV-2 testing and COVID-19 history information for the main and confirmatory outreach samples. No significant differences were found between samples, except for a higher asymptomatic proportion in the confirmatory sample.

#### CHR

The proportion of all confirmed COVID-19 cases hospitalized for COVID-19 was 9.4% (36/385; 95%CI: 6.6–12.7%). Among a subset of COVID-19 cases identified via the seroprevalence survey, the respective proportion was lower, at 5.5% (12/219; 95%CI: 2.9–9.4%). This estimate can be interpreted as an approximation of the infection hospitalization ratio (IHR), which is naturally lower than the CHR.

#### CFR and HFR

A total of 10 deaths occurred in the merged study sample during the observation period. Of these, 8 deaths were participants with confirmed COVID-19, of which 7 deaths were associated with COVID-19. One patient had confirmed COVID-19 and recovered, as confirmed by a negative PCR test, but died soon after recovery from terminal cancer. Of the 7 patients with COVID-related deaths, 3 were hospitalized, and 4 died rapidly around the time of diagnosis without hospitalization. The CFR among all confirmed COVID-19 cases was 1.8% (7/385; 95%CI: 0.7–3.7%), and the HFR was 8.3% (3/36; 95%CI: 1.8–22.5%).

### Dates of symptom onset

**Fig 1:**
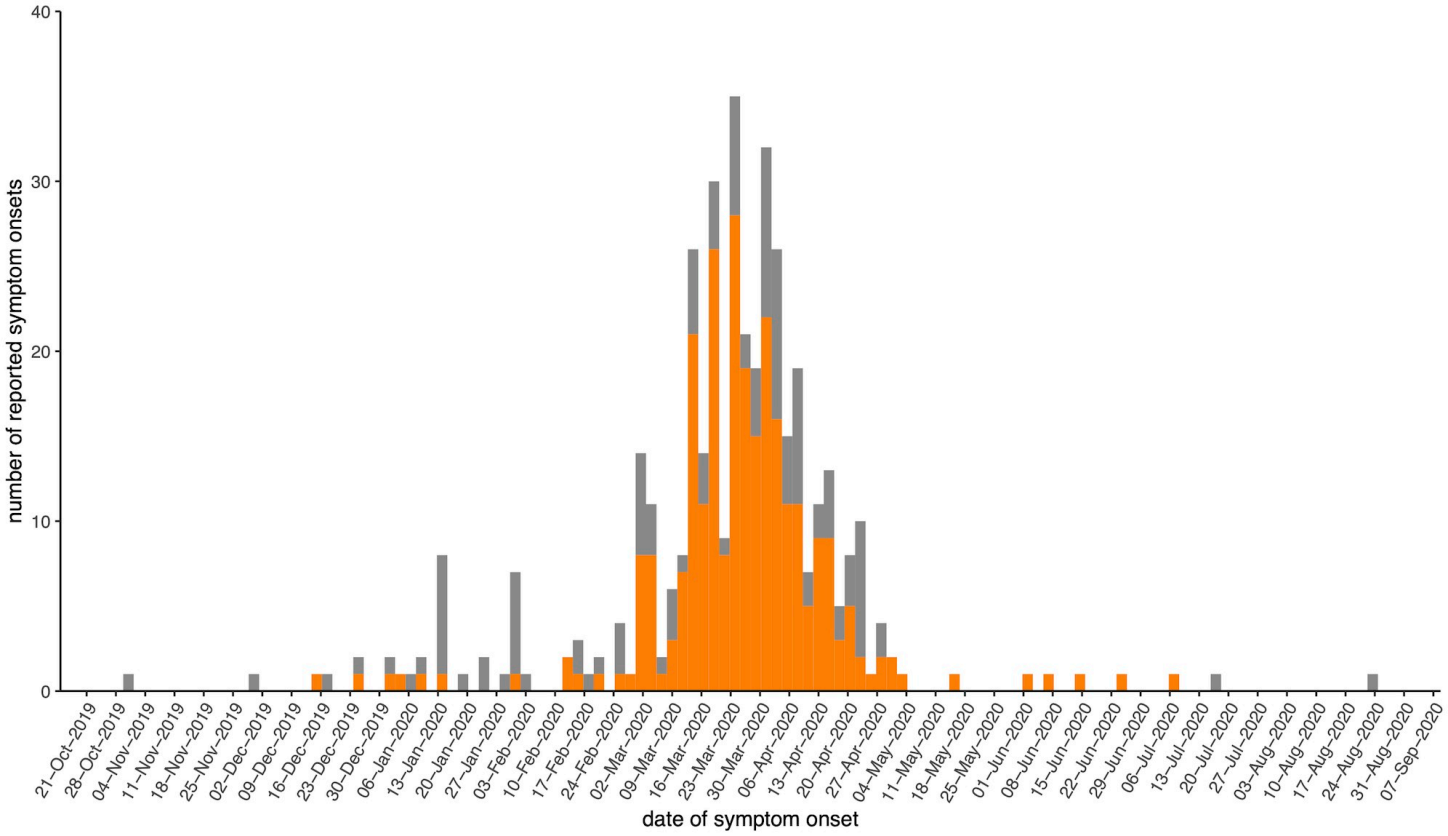
Dates of symptom onset according to COVID-19 status among the World Trade Center responder cohort in Long Island, NY between November 2019 and August 2020. Confirmed positive cases are shown in orange (N = 273), and unconfirmed cases are shown in gray (N = 129). Symptomatic cases confirmed to be negative for SARS-CoV-2 are not shown.

The symptom onset dates histogram shown in Fig 1 suggests that most cases occurred during the initial outbreak period. Although the first case in Long Island was officially reported on March 5^th^, these results suggest that some infections might have occurred as early as January and that several cases were present in late February. Similarly to the regional case count trajectory, the curve demonstrated an exponential increase in March and a rapid decrease starting approximately 2 weeks after the shutdowns were initiated. The curve followed a similar pattern among confirmed (orange) and unconfirmed (gray) symptomatic cases during the spring and summer. During the winter, when testing was not widely available, most symptomatic cases were classified as unconfirmed. Some of them might have been associated with influenza or other respiratory infections.

### Correlates of infection

Table 3 shows results of multivariable logistic regression to identify correlates of confirmed infection. Confirmed positive COVID-19 was significantly associated with younger age, being black or Hispanic vs. non-Hispanic white, being currently employed, and being prescribed ibuprofen. Being multiracial/other vs non-Hispanic white was negatively associated with confirmed COVID-19.

**Table 3:** Multivariable logistic regression: Correlates of confirmed SARS-CoV-2 infection among World Trade Center responders with known COVID-19 status (event N/total N: 385/1,202)^a^.

Characteristic ^b^	aOR	95% CI	p-value
Age (years)	0.97	(0.95–0.99)	0.0016
Sex			
Male	Ref		
Female	1.26	(0.82–1.94)	0.2962
Race / ethnicity			
Non-Hispanic white	Ref		
Black	1.81	(1.06–3.11)	0.0307
Hispanic	1.93	(1.08–3.43)	0.0260
Other / multiracial	0.64	(0.42–0.97)	0.0378
Currently working	2.00	(1.51–2.63)	<0.0001
Hypertension	1.63	(0.77–3.47)	0.2049
Diabetes	1.70	(0.59–4.92)	0.3247
Cancer	0.98	(0.71–1.34)	0.8869
Obstructive airway disease	1.08	(0.81–1.44)	0.6082
Upper respiratory disease	0.93	(0.68–1.28)	0.6599
Morbid obesity	1.19	(0.82–1.72)	0.3669
Depression	1.50	(0.81–2.75)	0.1947
Post-traumatic stress disorder	1.02	(0.67–1.54)	0.9333
Anxiety	0.81	(0.50–1.34)	0.4160
Prescribed ibuprofen	2.20	(1.00–4.83)	0.0499
Prescribed ACE inhibitors	1.68	(0.98–2.87)	0.0595
Prescribed statins	0.98	(0.69–1.40)	0.9277
Prescribed steroids	0.92	(0.68–1.26)	0.6135

aOR, adjusted odds ratio; CI, confidence interval; ACE, angiotensin-converting enzyme.

^a^ Regression analysis includes a subsample of confirmed COVID-19 positive (N = 385) and negative (N = 817) cases; unconfirmed cases are excluded.

^b^ This analysis did not use any automated subset selection methods; the table lists all candidate variables, which were included in the multivariable regression.

## Discussion

In this study, we conducted an investigation of COVID-19 epidemiology in a cohort of middle-aged men and women who responded to 9/11/2001 WTC events and resided in Long Island, NY. We used an array of data collection and analytic tools intended to minimize the bias and provide estimates of key epidemiologic characteristics in this population.

We estimated that approximately 22% of WTC responders were infected with SARS-CoV-2 from January to August of 2020. Our estimate of cumulative incidence is consistent with that previously reported in Long Island as of March 29, 2020, which was based on a convenience sample and indicated evidence of prior infection among 13.2% of study participants [3].

Our estimate of cumulative incidence is based on a seroprevalence survey among a consecutive sample of WTC responders presenting for health monitoring visits, corrected on the basis of available PCR testing results in the sample, and standardized by age, race/ethnicity, and employment status. Whereas significant differences in these demographic characteristics were observed between the seroprevalence survey sample and the rest of the cohort, standardization resulted in a very small change in the cumulative incidence estimate compared to the crude sample proportion, thus suggesting that the bias of the seroprevalence survey sample is likely to be minimal. However, unmeasured confounding might still be present. One potential source of confounding may be associated with the study participants’ knowledge of prior infection, which might have been an additional motivation to present for routine health monitoring visit rather than postponing or canceling, in which case the cumulative incidence would have been overestimated. Potential sources that could lead to the underestimation of cumulative incidence include lower than reported test sensitivity and the potential for rapid degradation of antibodies (in fewer than 4 months) among asymptomatic individuals, both of which are unlikely. Similarly to other studies (e.g., [40]), we found lower antibody levels in asymptomatic than symptomatic infections, although the difference was not statistically significant.

Although we found that older age was associated with a lower probability of infection, this phenomenon is likely to be a behavioral rather than biologic. Given that many WTC responders are essential workers, including police officers, exposure to infection in this cohort was probably comparable to, or somewhat higher than, that in the general population. Yet, we also found a higher PCR-based case ascertainment proportion (57%) among seroprevalence survey participants than what had been estimated in the general population [4], which may potentially be associated with regular testing requirements at work and better access to health care among many cohort members, as well as better testing availability in late spring and summer of 2020 than in early spring.

We estimated that approximately 16% of the SARS-CoV-2 infections in this population were asymptomatic. This estimate is consistent with the 17.9% estimated from an outbreak on the Diamond Princess cruise ship, in which the population was comparable in age to the WTC cohort [41]. This estimate can be interpreted as a lower bound for the true asymptomatic proportion in this population, owing to the potential unmeasured confounders discussed above, thus helping to narrow the range of currently available estimates—a topic of extensive scientific debate [22, 42].

Our estimates of CHR, CFR, and HFR are consistent with previously reported values, adjusting for age when available [15, 23, 25, 43–47]. This finding was somewhat unexpected, given that the population predominately comprised men with at least one comorbid condition and a high prevalence of pre-existing respiratory conditions; however uncertainty regarding CFR and HFR is high. Many positive cases in this study were confirmed with serology rather than PCR alone, thus potentially explaining lower CHR and CFR among confirmed cases [48]. At the same time, whereas 7 COVID-19 related deaths were reported, only 3 of those who died had previously been hospitalized, thus suggesting that one possible source for under-reporting of COVID-related mortality may have emerged if hospitals were considered central clearing houses for COVID-related deaths, particularly during the first wave of the pandemic [49]. Recognizing that COVID-19 might have caused many deaths before the implementation of the present study, we examined all death events in the parent cohort between March and August of 2020. The crude COVID-19 related mortality rate was slightly higher among the survey participants than the rest of the cohort, thereby suggesting that the study sample was unlikely to have been affected by survivor bias.

In the available sample, confirmed SARS-CoV-2 infection was correlated with demographic characteristics, including younger age, black or Hispanic race / ethnicity, and being currently employed rather than unemployed or retired. These associations are likely to be proxy measures for greater exposure probability. In our sample, none of the pre-existing conditions were associated with SARS-CoV-2 infection. We found that ibuprofen prescription was correlated with SARS-CoV-2 infection. Ibuprofen usage has been previously reported to be associated with greater COVID-19 severity [50], although the interpretation of this association remains unclear [51]. Further replication analyses are needed to determine whether these results are generalizable to other populations.

This is the first study of its kind in WTC responders, and one of the first studies to report results from a large non-hospital cohort globally. While this study avoids many limitations inherent to hospital-based cross-sectional or cohort studies, several limitations should still be noted. Although we made every effort to reach cohort members, approximately one-third of those contacted did not respond to requests for information. We addressed this issue by recruiting a confirmatory sample to correct for potential self-selection bias. A seroprevalence survey, which provided a basis for population-level estimates, was conducted over several months. Despite the similarity between the crude and standardized estimates of cumulative incidence, our results might still have unmeasured confounding. At the same time, antibody testing has been demonstrated to be more reliable than PCR testing in identifying infections. If residual confounding was present, it was likely to have been in the direction of overestimating the cumulative incidence and underestimating the asymptomatic proportion. Additional outreach efforts used to identify confirmed COVID-19 cases allowed for higher precision in the estimation of disease progression characteristics and assessment of the correlates of SARS-CoV-2 infection among WTC responders.

## Conclusions

In this study, we sought to investigate the extent to which COVID-19 affected middle-aged individuals in a hard-hit part of New York State through the end of summer 2020. We found a cumulative incidence of approximately 22% and a possibly earlier community transmission than has been generally accepted in Long Island, NY. Higher infection risk was associated with employment and younger age–variables likely to indicate higher exposure probability. We also found that black and Hispanic individuals had a higher risk of contracting the virus than non-Hispanic white individuals. None of the pre-existing conditions that we investigated were associated with increased infection risk.

## Supporting information

S1 Appendix Detailed description of the sample, estimation methodology, descriptive statistics of the outreach surveys, additional regression analysis results, and survey instruments. (PDF)
