# Supplementary material for: COVID-19 cumulative incidence, asymptomatic infections, and fatality in Long Island, NY, January–August 2020: A cohort of World Trade Center responders
Source: PLoS One. 2021 Jul 20;16(7):e0254713. doi: 10.1371/journal.pone.0254713 (PMC8291663; doi:10.1371/journal.pone.0254713)
Supplement: S1 Appendix — (PDF) [file pone.0254713.s001.pdf]

S1 Appendix to "COVID-19 cumulative incidence, asymptomatic infections, and fatality in Long Island, NY, January – August 2020: a cohort of World Trade Center responders"

Authors: Olga Morozova; Sean A. P. Clouston; Jennifer Valentine; Alexander Newman; Melissa Carr; Benjamin J. Luft

1. Outreach survey sample composition

The main outreach study sample (N = 6,093) is composed of participants of multiple outreach efforts:

- Telehealth visits: N = 1,053
- Brief needs assessment (BNA) survey: N = 4,665
- Brief text based (BTB) survey: N = 3,417
- Other (primarily from in-person monitoring visits, scheduling calls and electronic medical records; non-overlapping with other outreach efforts or seroprevalence survey): N = 53

The following diagram provides details about the overlap between participants of the first three survey modalities:

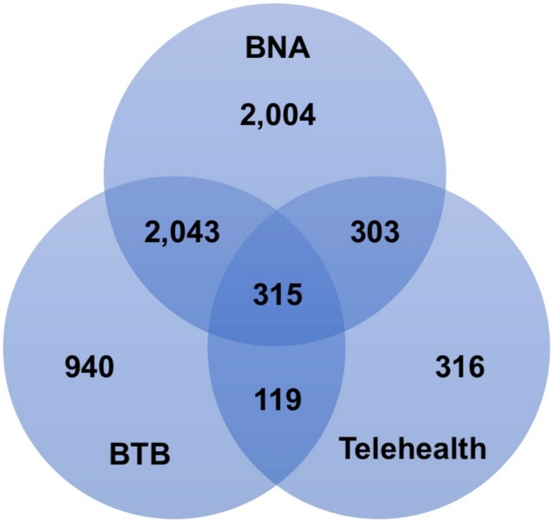

The following table shows details about the overlap between different survey modalities and the seroprevalence survey sample:

Survey modality	Seroprevalence survey participant		Total
	Yes	No	
Telehealth	297	756	1,053
BNA	593	4,072	4,665
BTB	499	2,918	3,417
Other	0	53	53

The BNA survey was repeated six times between March 16th and June 2nd with the following distribution of participation in multiple waves:

Number of BNA waves	Number of participants
6	29
5	76
4	319
3	711
2	1,335
1	2,195
Total	4,665

2. Selection bias correction based on confirmatory outreach survey sample

Let x be an epidemiologic parameter of interest (i.e. asymptomatic proportion), and let $\hat{x}$ be a point estimate of this parameter corrected for self-selection bias in the main outreach sample:

$$\hat{x} = \hat{x}_{main} \frac{N_{main}}{N} + \hat{x}_{conf} \left(1 - \frac{N_{main}}{N}\right), \qquad (1)$$

where $\hat{x}_{main}$ and $\hat{x}_{conf}$ are estimates of x from the main and confirmatory outreach samples respectively, N_{main} is the observed main outreach sample size, and N is the target population size (WTC responder cohort). The bias correction formula (1) is based on the population-level parameter estimation under stratified sampling approach [1].

3. Descriptive statistics of the main and confirmatory outreach samples

Table S1 shows demographic characteristics and Table S2 shows SARS-CoV-2 testing and COVID-19 history of the main and confirmatory outreach samples of the WTC responder cohort.

Table S1. Demographic characteristics of the main outreach sample (N=6,093) and the confirmatory outreach sample (N=255) of the World Trade Center responder cohort

	Main outreach sample (N=6,093 [a])		Confirmatory sample (N=255 [a])		
Characteristic	N	%	N	%	p-value [b]
**Age, years old:** mean (SD)	56.8 (8.1)		54.5 (8.1)		< 0.0001
**Age, categories, years old**					
30-39	25	0.4%	2	0.8%	0.0010
40-49	1065	17.5%	71	27.8%	
50-59	2943	48.3%	124	48.6%	
60-69	1596	26.2%	42	16.5%	
70-79	432	7.1%	14	5.5%	
80+	31	0.5%	2	0.8%	
**Sex**					
Male	5497	90.2%	237	92.9%	0.1605
Female	596	9.8%	18	7.1%	
**Race / Ethnicity**					
Non-Hispanic white	5068	83.2%	213	83.5%	0.9922
Black	335	5.5%	14	5.5%	
Hispanic	363	6.0%	14	5.5%	
Other / multiracial / unknown	327	5.4%	14	5.5%	
**Employment**					
Employed	3175	52.1%	157	61.6%	0.0523
Retired	2547	41.8%	87	34.1%	
Unemployed / laid off	73	1.2%	1	0.4%	
On extended leave or disability	239	3.9%	8	3.1%	
Other / unknown	59	1.0%	2	0.8%	

[a] numbers may not sum up to totals due to missing values and percentages may not sum up to 100 due to rounding.
[b] p-value for independent sample t-test or Pearson's Chi-squared test, simulated using bootstrap with 1,000,000 iterations.

Table S2. SARS-CoV-2 testing and COVID-19 history and status in the main outreach sample (N=6,093) and the confirmatory outreach sample (N=255) of the World Trade Center responder cohort

	Main outreach sample (N=6,093 [a])		Confirmatory sample (N=255 [a])		
Characteristic	N	%	N	%	p-value [b]
**SARS-CoV-2 PCR testing**					
Positive	265	4.3%	9	3.5%	0.3715
Negative	76	1.2%	1	0.4%	
No reported PCR testing	5752	94.4%	245	96.1%	
**SARS-CoV-2 IgG antibody testing**					
Positive	182	3.0%	5	2.0%	0.2269
Negative	588	9.7%	18	7.1%	
No reported antibody testing	5323	87.4%	232	91.0%	
**COVID-19 status**					
Confirmed positive	341	5.6%	12	4.7%	0.3434
Confirmed negative	582	9.6%	18	7.1%	
Symptomatic, PCR-, no antibody test	43	0.7%	0	0.0%	
Symptomatic, never tested	111	1.8%	4	1.6%	
No reported symptoms or testing	5016	82.3%	221	86.7%	
**COVID-19 case classification among confirmed positive**					
Asymptomatic	24	7.0%	4	33.3%	0.0505
Mildly symptomatic	278	81.5%	7	58.3%	
Severe	38	11.1%	1	8.3%	
Unknown	1	0.3%	0	0%	
**Hospitalized for COVID-19 among confirmed positive**					
Yes	34	10.0%	1	8.3%	>0.9999
No	307	90.0%	11	91.7%	
**Deceased among confirmed positive**					
Yes, COVID-19 related	7	2.1%	0	0.0%	>0.9999
Yes, other reasons	1	0.3%	0	0.0%	
No	333	97.7%	12	100.0%	
**Deceased among hospitalized for COVID-19**					
Yes	3	8.8%	0	0.0%	>0.9999
No	31	91.2%	1	100.0%	

[a] numbers may not sum up to totals due to missing values and percentages may not sum up to 100 due to rounding.
[b] p-value for independent sample t-test or Pearson's Chi-squared test, simulated using bootstrap with 1,000,000 iterations.

4. Correlates of SARS-CoV-2 infection: Additional regression analysis

Table S3 shows regression analysis results with age included as a categorical (rather than continuous) variable.

Table S3. Multivariable logistic regression: Correlates of confirmed SARS-CoV-2 infection among WTC responders with known COVID-19 status (event N / total N: 385 / 1,202)[a]

Characteristic	aOR	95% CI	p-value
Age, categories, years old			
Less than 50	Ref		
50-59	0.64	(0.46 - 0.89)	0.0076
60-69	0.49	(0.33 - 0.74)	0.0006
70+	0.51	(0.27 - 0.96)	0.0376
Sex			
Male	Ref		
Female	1.25	(0.81 - 1.94)	0.3084
Race / Ethnicity			
Non-Hispanic white	Ref		
Black	1.85	(1.07 - 3.17)	0.0269
Hispanic	2.00	(1.12 - 3.56)	0.0196
Other / multiracial	0.64	(0.42 - 0.98)	0.0392
Currently working	2.02	(1.53 - 2.66)	<0.0001
Hypertension	1.61	(0.76 - 3.43)	0.2138
Diabetes	1.62	(0.57 - 4.62)	0.3699
Cancer	0.96	(0.70 - 1.32)	0.8192
Obstructive airway disease	1.08	(0.81 - 1.44)	0.6113
Upper respiratory disease	0.94	(0.68 - 1.29)	0.6931
Morbid obesity	1.19	(0.82 - 1.73)	0.3573
Depression	1.56	(0.84 - 2.87)	0.1574
Post-traumatic stress disorder	1.00	(0.66 - 1.52)	0.9899
Anxiety	0.80	(0.49 - 1.31)	0.3736
Prescribed ibuprofen	2.19	(1.00 - 4.82)	0.0505
Prescribed ACE inhibitors	1.71	(1.00 - 2.92)	0.0508
Prescribed statins	0.99	(0.69 - 1.40)	0.9357
Prescribed steroids	0.94	(0.68 - 1.28)	0.6739

aOR, adjusted odds ratio; CI, confidence interval

[a] Regression analysis includes a subsample of confirmed COVID-19 positive (N=385) and negative (N=817) cases; unconfirmed cases are excluded.

5. Survey instruments

5.1 Telehealth and in-person visits

Information was entered in the study database based on chart reviews containing standard monitoring visit instruments and clinical notes, as well as linked electronic medical records and laboratory records.

5.2 Brief Needs Assessment (BNA)

To what extent has the Coronavirus impacted:

	Not at all	A little	A great deal	N/A
Your daily routine	○	○	○	○
Your family relationships	○	○	○	○
Your quality of life	○	○	○	○
Your mental well-being	○	○	○	○
Your physical well-being	○	○	○	○
Your sleep	○	○	○	○
Keeping/making appointments with the WTC clinic	○	○	○	○
Keeping/making other medical appointments	○	○	○	○

How much do you agree with the following statements?

	Strongly Disagree	Disagree	Undecided	Agree	Strongly Agree	N/A
I worry that my risk of exposure is high	○	○	○	○	○	○
If exposed, I worry that my risk of developing a serious illness is high	○	○	○	○	○	○
The situation has affected my current mental health conditions	○	○	○	○	○	○
The situation has affected my current physical health conditions	○	○	○	○	○	○

Where do you prefer to get updates about the virus? Please check all that apply.

- ☐ TV or radio
- ☐ Newspapers
- ☐ World Trade Center Health Program
- ☐ Medical professionals
- ☐ State or federal health departments or CDC

Have you or someone you know been tested and diagnosed by a health professional with COVID-19? Please check all that apply.

- ☐ I was diagnosed and hospitalized
- ☐ I was diagnosed and recovered at home
- ☐ Someone in my household was diagnosed and hospitalized
- ☐ Someone in my house was diagnosed and recovered at home
- ☐ A neighbor or friend was diagnosed and hospitalized
- ☐ A neighbor or friend was diagnosed and recovered at home
- ☐ I don't know anyone who's been diagnosed with COVID-19

Date diagnosed:

____

Date hospitalized:

____

During this time are you:

- ☐ Working outside the home around people
- ☐ Self-isolating and living alone
- ☐ Self-isolating and living with others
- ☐ Currently in quarantine

Please share with us any comments or feedback you may have about COVID-19, your WTC-conditions (physical or psychological), or any other concerns.

____

____

____

____

____

5.3 Brief text-based survey (BTB)

Have you been infected with COVID-19?

○ Yes

○ No

Have you been tested for COVID-19?

○ Yes

○ No

5.4 Follow-up survey

1. How have you been feeling?
2. Do you need any assistance at this moment? (e.g. medication refills, case management, mental health counseling, etc.)
3. Do you have any questions/concerns regarding COVID-19?
4. When did your symptoms begin?
   a. MM/DD/YY
   b. I never experienced symptoms
5. Compared to yesterday, do you feel the same, better or worse?

6. Symptoms Checklist: Severity of Symptoms
1-Absent, 2-Mild, 3-Moderate, 4-Severe

Fever?	Score:		
If yes, what was your highest temperature in the last 24 hours? ______			
Sweats?	Score:	Shortness of Breath (SOB)?	Score:
Cough?	Score:	Sore Throat?	Score:
Pain in the chest?	Score:	Chills?	Score:
Muscle/Body Aches?	Score:	Headaches?	Score:
Diarrhea?	Score:	Fatigue?	Score:
Have you lost your sense of smell?		Score:	
OTHER?		Score:	

7. Is patient having trouble speaking in full sentences? Y N

8. Were you exposed to anyone tested and diagnosed by a health professional with COVID-19?
   a. If so, who and when?

9. Have you travelled recently?
   a. Where and when?

If applicable, confirm patient's WTC-certified conditions (reference EMR)

10. Do you have any cardiovascular diseases, respiratory conditions, diabetes, hypertension and/or immunosuppression?

11. Are you currently working or doing volunteer work out of the house?
    If yes,
    i. What is your occupation title?
    ii. What is the zip code/town that you work in?
    iii. Are you working around other people?
    iv. Are you keeping a safe distance from others?
    v. Are you wearing a face mask?

12. What zip code are you currently living in?

13. Have you been in contact with your primary care provider (PCP)?
    If yes, did he/she recommend you to get testing?

14. Have you been tested?
    If yes,
    i. Where and when were you tested?
    ii. What were your results? Negative, Positive, Pending
    iii. Are you currently self-quarantined?
    iv. Were you prescribed hydroxychloroquine?
    v. Were you prescribed z-pack?

vi. Other treatments?

15. Did you go to an ER for COVID symptoms?
    a. Were you hospitalized?
    b. Where and when were you treated?
    c. How were you treated?
        i. Hydroxychloroquine?
        ii. Z-pack?
        iii. Ventilator?
        iv. Oxygen?
        v. Other?

    d. Were you admitted to an ICU?

    e. When were you discharged? (MM/DD/YY)

16. <u>Caller Notes:</u>

17. <u>Referrals Made:</u>

References

[1] Thompson SK. Sampling. 3rd ed. Hoboken, New Jersey: John Wiley and Sons, Inc; 2012.
